# Cerebellar glioblastoma in adults: a comparative single-center matched pair analysis and systematic review of the literature

**DOI:** 10.1007/s00432-024-05959-0

**Published:** 2024-09-28

**Authors:** Yauhen Lizunou, Anna-Laura Potthoff, Niklas Schäfer, Andreas Waha, Valeri Borger, Ulrich Herrlinger, Hartmut Vatter, Patrick Schuss, Matthias Schneider

**Affiliations:** 1 Department of Neurosurgery, University Hospital Bonn, Bonn, Germany; 2 Department of Neurology, Devision of Neurooncology, University Hospital Bonn, Bonn, Germany; 3 Department of Neuropathology, University Hospital Bonn, Bonn, Germany; 4 Department of Neurosurgery, Unfallkrankenhaus Berlin, Berlin, Germany

**Keywords:** Cerebellar glioblastoma, Supratentorial glioblastoma, Matched pair analysis, Overall survival, Propensity score matching

## Abstract

**Purpose:**

The rarity of cerebellar glioblastoma presents a significant challenge in clinical practice due to the lack of extensive prognostic data on long-term survival rates, rendering it an underrepresented entity compared to its supratentorial counterpart. This study aims to analyze potential differences in survival outcome between patients with cerebellar and supratentorial glioblastomas.

**Methods:**

From 2009 to 2020, 8 patients underwent surgical treatment for cerebellar glioblastoma at the authors’ institution. These patients were individually matched with a cohort of 205 consecutive patients from our institutional database with supratentorial glioblastoma, taking into account key prognostic parameters. Progression-free survival (PFS) and overall survival (OS) rates were compared. Additionally, we performed a systematic literature review to compile further survival data on cerebellar glioblastoma patients.

**Results:**

The median OS for cerebellar glioblastoma patients was 18 months (95% CI 11–25). The balanced matched-pair analysis showed no significant difference in survival when compared to patients with supratentorial glioblastoma, exhibiting a median OS of 23 months (95% CI 0–62) (*p* = 0.63). Respective values for PFS were 8 months (95% CI 4–12) for cerebellar and 7 months (95% CI 0–16) for supratentorial glioblastoma (*p* = 0.2). The systematic review revealed that median OS for cerebellar glioblastoma in current literature ranges from 7 to 21 months.

**Conclusions:**

The present findings indicate that patients with supra- and infratentorial glioblastoma do not significantly differ in regard to survival outcome parameters. This similarity in prognosis might encourage clinicians to consider surgical interventions for both supra- and infratentorial glioblastoma in a similar manner.

**Supplementary Information:**

The online version contains supplementary material available at 10.1007/s00432-024-05959-0.

## Introduction

Glioblastoma ranks among the most aggressive primary malignant brain tumors in adults (Ostrom et al. 2020). Numerous studies have been undertaken to hasten the development of more advanced treatment strategies through diverse therapeutic and surgical methods (Potthoff et al. 2019; Schneider et al. 2019, Venkataramani et al. 2022; Dejonckheere et al. 2023). Glioblastomas are most commonly found in the supratentorial region, with less than 1% occurring in the cerebellum (Tsung et al. 2011). Previous research has highlighted distinct molecular and clinical profiles between supratentorial and cerebellar glioblastomas, suggesting that cerebellar glioblastoma may represent a distinct subgroup (Adams et al. 2013; Hong et al. 2018). Nonetheless, survival data on cerebellar glioblastoma remain sparse, and existing reports often rely on extended historical data sets and varied therapeutic approaches, which may affect consistency (Adams et al. 2013; Levine et al. 1987).

In response to this gap, we analyzed institutional data on adult cerebellar glioblastoma and conducted a matched-pair analysis to draw comparisons with supratentorial glioblastoma patients treated at our facility. Additionally, we conducted a systematic review of the literature following the PRISMA guidelines (Page et al. 2021), extracting individual patient data to compare our findings with the broader corpus of clinical research on this rare form of glioblastoma.

## Methods

### Patients

Patients aged 18 years or older with newly diagnosed cerebellar glioblastoma who underwent surgical intervention at our institution between 2009 and 2020 were systematically entered into a computerized database (SPSS, version 27, IBM Corp., Armonk, NY). Our study adhered to the principles of the Helsinki Declaration and received approval from the institutional ethics committee (228/19). Informed consent was not sought in regard to the retrospective study design. Data collection occurred from 2020 to 2021, during which all relevant patient information was systematically entered into the database.

We meticulously recorded data, including patient demographics, radiological findings, isocitrate dehydrogenase (IDH)-status, O6-methylguanine-DNA methyltransferase (MGMT) promoter methylation status, and functional neurological status objectified by the Karnofsky performance scale (KPS) at both admission and throughout the course of treatment as previously described (Schneider et al. 2020, Borger et al. 2021; Schneider et al. 2021; Schneider et al. 2020). The institutional interdisciplinary tumor board made treatment decisions encompassing the extent of neurosurgical intervention and postoperative care (Schäfer et al. 2021).

Tumor volumes were volumetrically assessed based on MRI T1 with contrast enhancement.

The extent of resection (EOR) was determined based on early postoperative 3.0-Tesla MRI scans conducted within 72 h following surgery. Definitions of gross-total resection (GTR) and subtotal resection (STR) corresponded to the removal of more than 95% and less than 95% of the initial contrast-enhancing tumor tissue, respectively (Shonka and Aizenberg 2017; Schneider et al. 2019). We excluded patients who had subtotal resection, partial resection or only a biopsy, as well as those with additional tumor infiltration into the brainstem, due to the known poorer prognosis (Kreth et al. 2013; Simpson et al. 1993; Weber et al. 2006). We also collected data on the use of 5-aminolevulinic acid (ALA)-guided surgery at our institution.

Operative complications were meticulously documented. Postoperative complications were defined as any adverse event occurring within 30 days following the initial glioblastoma resection that necessitated further intervention and/or surgery.

Following histopathological confirmation of glioblastoma, MGMT promoter methylation status was ascertained using pyrosequencing and combined bisulfite restriction analysis (Mikeska et al. 2007). Tumor classification followed the 2016 WHO classification criteria.

Progression-free survival (PFS) was defined as the interval from glioblastoma surgery to the date of radiological progression, as previously outlined (Zeyen et al. 2023). Tumor progression criteria followed the Response Assessment in Neuro-Oncology (RANO) guidelines with certain modifications (Herrlinger et al. 2019).

Overall survival (OS) was calculated from the date of glioblastoma surgery until the patient’s death. Post-surgical treatment for all patients included adjuvant therapies according to the Stupp- (Stupp et al. 2005) or CeTeG-protocol (Herrlinger et al. 2019) in line with the institutional interdisciplinary tumor board’s recommendations (Schäfer et al. 2021).

### Matching procedure

For the purpose of conducting a matched-pair analysis, the statistical computing software R was used (version 4.2.3; The R Foundation for Statistical Computing, available at [https://www.r-project.org/]). The cohort of consecutive individuals who had undergone resection of supratentorial glioblastoma at our university hospital between 2013 and 2018 comprised 205 patients. This patient cohort was matched with the cerebellar cohort of eight patients. We applied a multivariate approach and propensity score matching with an aim for balance optimization, setting a matching ratio of 1:4 as previously described (Hamed et al. 2022, 2023; Layer et al. 2023).

In our effort to enhance the balance between the matched pairs, we selected several generally known prognostic factors for matching. These factors included age, KPS at admission, EOR categorized as either GTR or STR, and MGMT promoter methylation status, as these have been identified as predictors on patient outcomes (Li et al. 2016; McGirt et al. 2009; Radke et al. 2019; Smrdel et al. 2016). Subsequently, from the pool of patients with supratentorial glioblastoma, we selected 32 individuals who, based on R software comparisons, were best matched to our cerebellar tumor cohort.

### Systematic review of the literature

#### Search methods

A systematic literature review was conducted to gather current published data on survival outcomes for cerebellar glioblastoma. We conducted a search on PubMed and the Cochrane Library, covering publications from 2005 to 2023. The systematic review of the literature was performed following PRISMA guidelines (Page et al. 2021). The search strategy included specific keywords: “cerebellar glioblastoma,” “posterior fossa glioblastoma,” and “infratentorial glioblastoma,” with the final search conducted on the 31 December 2023.

In the initial screening phase, the titles and abstracts were reviewed to assess relevance to our research question. Full-text versions of pertinent studies were then retrieved for detailed evaluation. Two authors (PS and MS) independently reviewed the selected articles to ensure comprehensive analysis. Discrepancies were resolved through a consensus meeting with the senior author (MS). Additionally, we examined the reference lists of identified articles to find further relevant studies published within the specified timeframe.

#### Selection criteria

Studies were included if they reported individual patient data on the treatment of patients with cerebellar glioblastoma. Exclusion criteria comprising studies focusing exclusively on pediatric patients, as well as studies describing treatment of cerebellar glioblastoma with supratentorial fraction and/or brainstem involvement. Only articles published in English or German were considered.

#### Data collection and extraction

If reported, the following data was extracted from qualifying articles: study design, number of patients with cerebellar glioblastoma, preoperative KPS, extent of resection, histopathological features, PFS, and OS. Not all studies provided data or information on each subset of patients. If included studies reported only insufficient data on the above-mentioned factors, these were excluded from further analysis. Useful data was independently extracted and verified by two authors (PS and MS). No disagreements were found.

### Statistics

Data analyses were performed using the computer software package SPSS (version 27, IBM Corp., Armonk, NY). Mann-Whitney U test was used for parametric statistics. Categorical variables were analyzed in contingency tables using Fisher’s exact test. Results with *p* < 0.05 were considered statistically significant. OS and PFS were analyzed by the Kaplan-Meier method. Events in survival curves were defined as radiographic tumor progression and death and compared by using the Gehan-Breslow-Wilcoxon test.

## Results

### Patient characteristics

Between 2009 and 2020, 8 patients underwent surgical therapy for cerebellar glioblastoma at the authors’ institution. The median age at the day of surgery was 70 years (IQR 64–76) and GTR was performed in 7 out of 8 patients (88%). The median tumor volume in our cohort was 14.5 cm³, with IQR of 10 to 30.25 cm³. 7 out of 8 cerebellar glioblastomas (88%) were located in the cerebellar hemispheres, with 1 (12%) involving the vermis. No cases of leptomeningeal dissemination were observed in our cohort. Postoperative treatment information was available for 6 out of 8 patients (75%), with 5 (63%) treated according to the Stupp protocol. One patient (12%) did not receive adjuvant postoperative therapy due to early postoperative death caused by a hemorrhage.

Of the eight patients studied, five were reclassified according to the new WHO guidelines as glioblastoma, IDH-Wildtype (CNS WHO Grade 4). Molecular analysis revealed methylated and unmethylated MGMT-promoter status in 5 (63%) and 3 (37%) patients. The median OS rate was 18 months (95% CI 11–25). For further details see Table 1.

**Table 1:** Patient characteristics in the present series of patients with cerebellar glioblastoma

Case No.	Age (yrs)	KPS pre	Tumor localization	EOR	MGMT*	IDH status (wt/mut)	PFS (m)	OS (m)
1	78	80	hemisphere	GTR	-	na	4	4
2	63	60	hemisphere	GTR	+	wt	12	20
3	35	80	hemisphere	GTR	+	wt	17	23
4	76	60	hemisphere	GTR	+	wt	3	3
5	70	70	hemisphere	GTR	+	wt	11	31
6	30	70	vermis	GTR	-	wt	7	49
7	76	60	hemisphere	STR	+	wt	1	1
8	67	40	hemisphere	GTR	-	wt	1	1

* ‘-‘, promoter unmethylated; ‘+’, promoter methylated

** (values represent number of patients unless indicated otherwise)

EOR, extent of resection; GTR, gross total resection; IDH, isocitrate dehydrogenase; KPS, Karnofsky performance scale; m, months; MGMT, O-6-methylguanine-DNA methyltransferase; mut, IDH-mutation, na, not available; No, number; OS, overall survival; PFS, progression-free survival; pre, preoperative at admission; STR, subtotal resection; wt, wildtype; yrs, years

### Comparative matched pair analysis reveals comparable survival rates for cerebellar and supratentorial glioblastoma

**Fig. 1:**
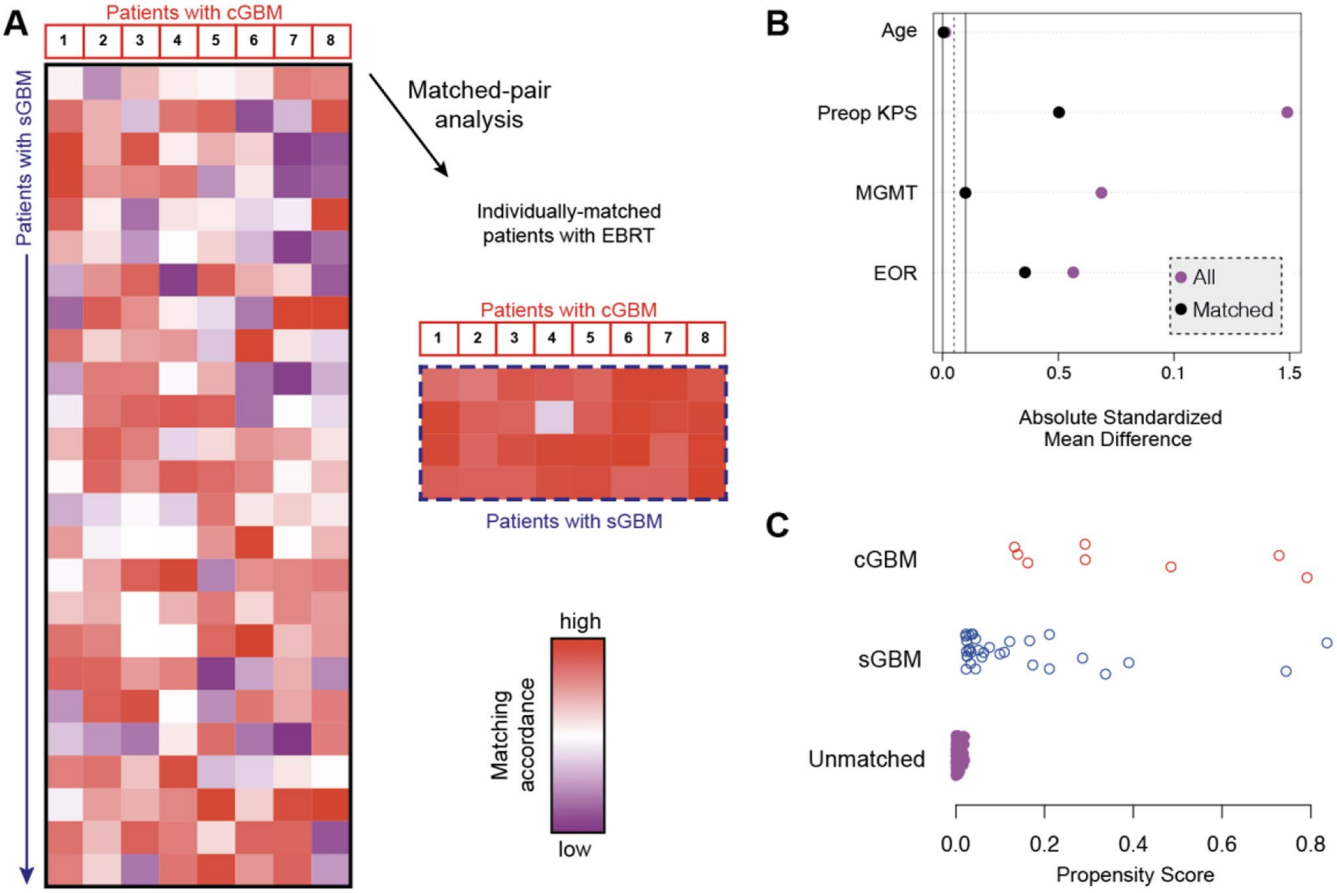
Illustration of matching procedure between cerebellar and supratentorial glioblastoma patients. (**A**) Comparative matched pair analysis at a ratio of 1:4 identifies 32 out of 205 patients with supratentorial glioblastoma that individually correspond to the present series of 8 patients with cerebellar glioblastoma. Heat map as color-coded illustration of the matching strategy of supratentorial glioblastoma to individually-matched cerebellar glioblastoma cases by means of age at admission, KPS at admission, EOR as well as MGMT promoter methylation status as matching parameters. Red frames depict individually-matched supratentorial glioblastoma patients. (**B**) Visualization of mean difference between patients with supratentorial glioblastoma (purple stands for all patients and black - for patients after matching). (**C**) Visualization of obtained propensity scores for matched and unmatched glioblastoma patients. EOR, extent of resection; KPS, Karnofsky performance scale; MGMT, O6-methylguanine-DNA methyltransferase

In order to compare median survival rates of the cohort of cerebellar glioblastoma patients and corresponding patients with supratentorial glioblastoma, we performed a multivariate and propensity score matching with additional balance optimization. Therefore, cerebellar glioblastoma patients were individually matched at a ratio of 1:4 to a cohort of 205 consecutive patients that had undergone resection of supratentorial glioblastoma at our university hospital between 2013 and 2018 (Fig. 1). Patient age and KPS at admission, EOR as well as MGMT promoter methylation status were chosen as matching parameters.

Hence, matched pair analysis yielded two individually matched cohorts of 8 cerebellar and 32 supratentorial glioblastoma patients that did not significantly differ with regard to above mentioned prognostic parameters (Table 2). Following the matching procedure, IDH status did not differ for the two groups of glioblastoma patients. In our study, 5-ALA-guided resection was performed in 3 out of 8 cerebellar glioblastoma patients (38%) and in 20 out of 32 (63%) supratentorial glioblastoma patients (*p* = 0.25). Postoperative treatment information was available for 6 out of 8 patients (75%), with 5 (63%) treated according to the Stupp protocol. One patient (12%) did not receive adjuvant postoperative therapy due to early postoperative death caused by a hemorrhage.

**Table 2:** Comparison of clinical and surgical outcomes in patients with cerebellar and supratentorial tumor localizations

	Cerebellar localization *n* = 8	Supratentorial localization *n* = 32	*p* Value
Number of patients	8	32	
Median age (years, IQR)	70 (64–76)	66 (55–74)	0.22
Median KPS at admission (IQR)	65 (60–70)	70 (60–80)	0.33
EOR (STR/GTR)	1/7	8/24	0.45
5-ALA-guided resection	3 (37.5)	20 (62.5)	0.25
IDH-Status (wt/mut)	7/-^Ω^	32/0	-
MGMT-promoter methylation	5 (62.5)	26 (81.5)	0.26
Median PFS (mo, 95% CI)	8 (4–12)	7 (0–16)	0.2
Median OS (mo, 95% CI)	18 (11–25)	23 (0–62)	0.63

* 7 patients due to 1 patient with missing IDH status

** (values represent number of patients unless indicated otherwise)

5-ALA, 5-Aminolevulinic acid, EOR, extent of resection; IDH, isocitrate dehydrogenase; GTR, gross total resection; KPS, Karnofsky performance scale; mo, months; MGMT, O-6-methylguanine-DNA methyltransferase; OS, overall survival; PFS, progression-free survival; STR, subtotal resection; yrs, years

For those with supratentorial glioblastoma, we observed following postoperative treatment-protocol distribution: 17 out of 32 patients (53%) were treated according to the Stupp protocol, 9 out of 32 (28%) received the CeTeG regimen, 3 out of 32 (9%) did not receive chemotherapy, and 3 out of 32 patients (9%) were placed on palliative care within the first three months following surgical resection, and thus did not complete adjuvant treatment protocols. There were no cases of re-operation in either the cerebellar cohort or the matched supratentorial cohort.

**Fig. 2:**
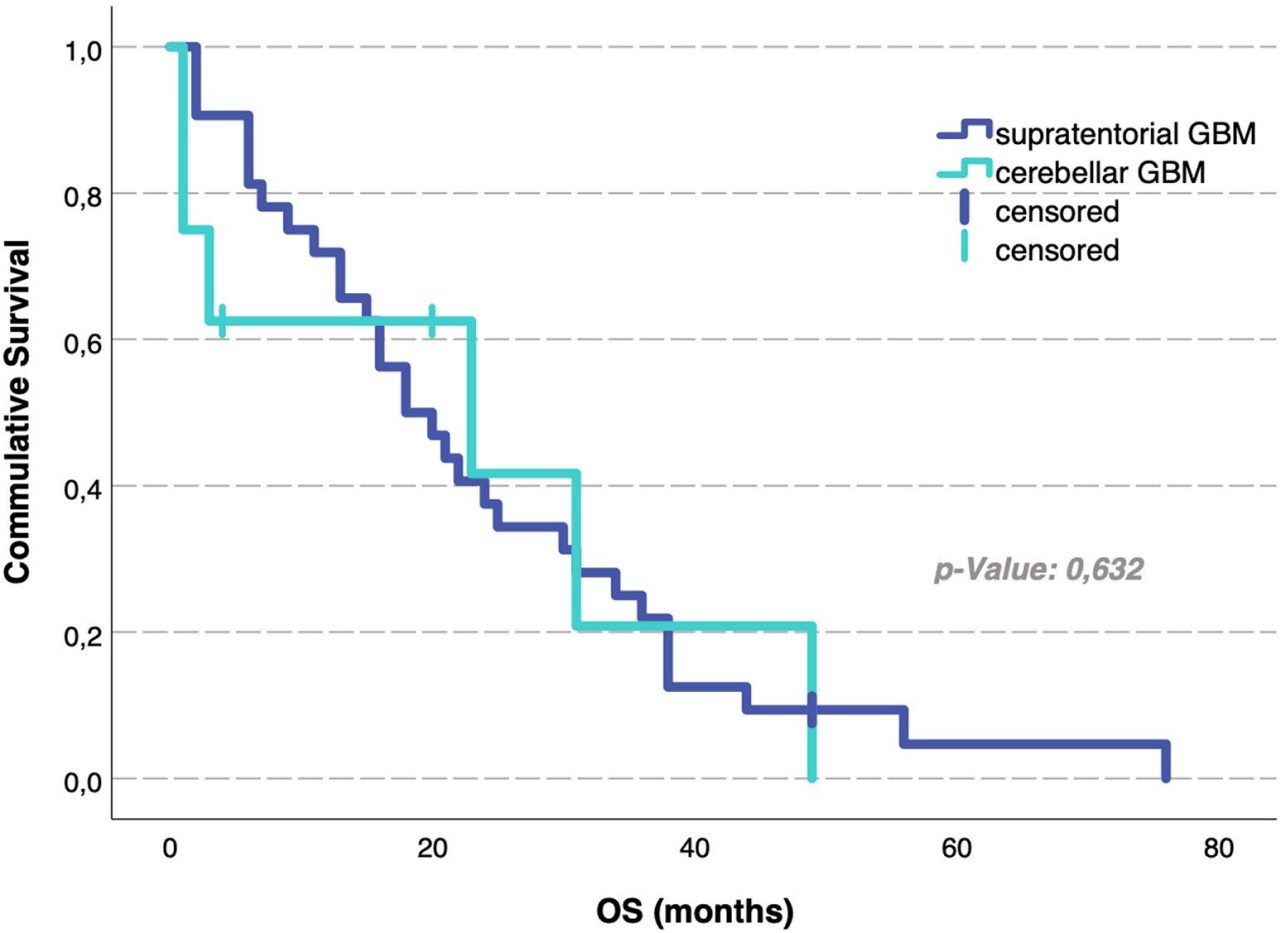
Overall survival analysis for cerebellar and individual matched supratentorial glioblastoma patients

**Fig. 3:**
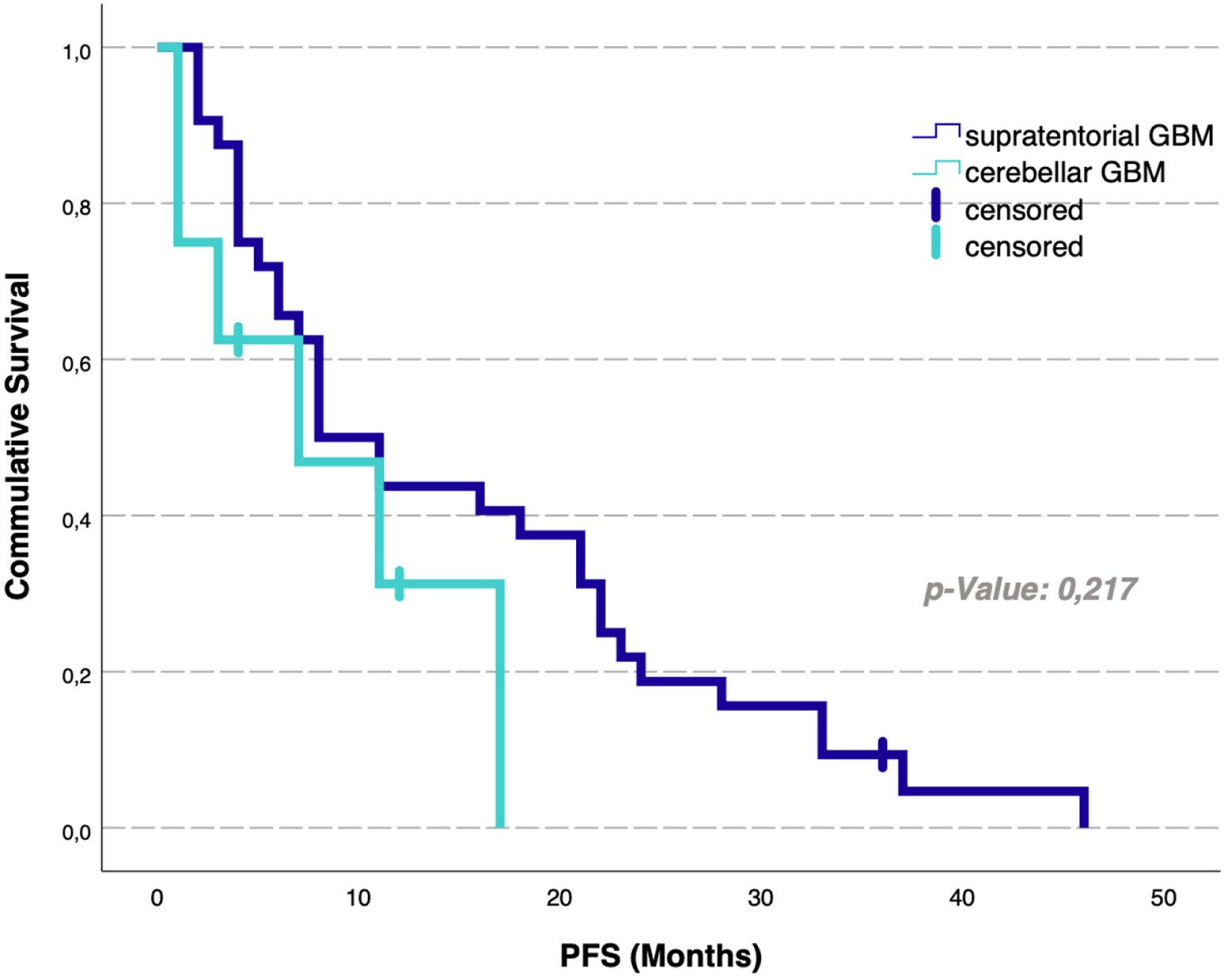
Progression-free survival analysis for cerebellar and individual matched supratentorial glioblastoma patients

Analysis of median OS rates revealed no statistically significant differences depending on the tumor localization: patients with cerebellar glioblastoma revealed an OS rate of 18 months (95% CI 11–25) compared to 23 months (95% CI 0–62) for individually matched supratentorial glioblastoma patients (*p* = 0.63) (Table 2; Fig. 2). PFS did also not significantly differ between the cerebellar cohort (8 months, 95% CI 4–12) and the supratentorial cohort (7 months, 95% CI 0–16), with *p* = 0.2 (Table 2; Fig. 3). For the total group of 205 patients with supratentorial glioblastoma, analysis of median OS rates yielded 17 months (95% CI 14,4–18,6).

### Systematic review of the literature concerning survival rates of patients with cerebellar glioblastoma

**Fig. 4:**
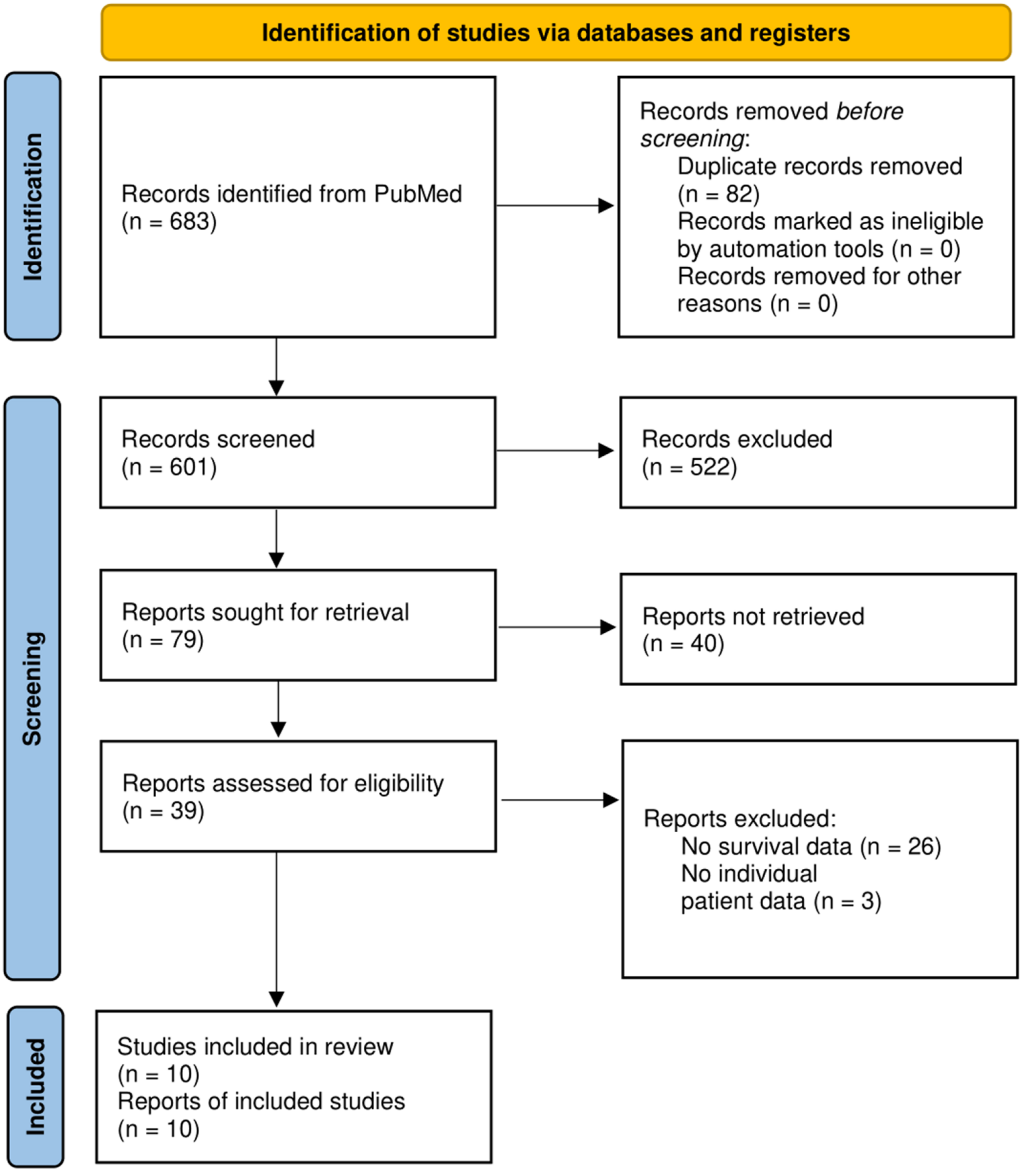
Flowchart depicting the search strategy for contemporary review of the literature regarding survival in cerebellar glioblastoma patients

The search of the literature on cerebellar glioblastoma from 2005 to 2023 yielded 709 articles (Fig. 4, Figure S1). Given that the clear assignment of PFS and/or OS to this specific patient cohort was the main inclusion criterion, a total of 10 studies were ultimately identified for further data analysis (Hong et al. 2018; Cho et al. 2019; Picart et al. 2018; Takahashi et al. 2014; Milinkovic et al. 2014; Gopalakrishnan et al. 2012; Utsuki et al. 2012; Broekman ML et al. 2009, Al-Barbarawi MM et al. 2009, Mattos JP et al. 2006). Median age of patients in these studies was 48.5 years (IQR 46-52.2). Median OS rates ranged from 7 to 21 months (Table 3). An important aspect of systematic reviews is the evaluation of the risk of bias. There are well-established tools for evaluating the risk of bias, such as those provided by the Cochrane Library (e.g., the ROBINS-I tool (2016). However, due to the heterogeneity of the included studies —ranging from large group studies to case reports — and the often incomplete data reported in these studies, it is not feasible to apply these tools comprehensively.

**Table 3:** Contemporary systematic review of the literature for cerebellar glioblastoma

First author (year)	Included in further analysis	Mean age (years)	Mean KPS pre	EOR (STR/GTR)	MGMT	Median PFS (m)	Median OS (m)
Hong et al. (2018)	6	48	na	3 / 3	5	3	7
Cho et al. (2019)	19	57	na	na	na	na	21
Picart et al. (2018)	14	53	na	na/8	3	4	8
Takahashi et al. (2014)	9	56	68	na/2	na	na	9
Milinkovic et al. (2014)	5	46	na	na/2	na	8	18
Gopalakrishnan et al. (2012)	4	50	92	0/4	na	10.4	9
Utsuki et al. (2012)	4	49	na	2 / 2	na	8	20.5
Broekman ML et al. (2009)	1	28	70	-/1	na	6	12
Al-Barbarawi MM et al. (2009)	1	41	60	-/1	na	14	15
Mattos JP et al. (2006)	1	46	60	1/-	na	na	18
Present series (2023)	8	68.5	65	1/7	5	8	18
Total	72	49.3	69	7/30	13	8	15

Values represent number of patients unless indicated otherwise

EOR, extent of resection; GTR, gross total resection; KPS, Karnofsky performance scale; m, months; MGMT, O-6-methylguanine-DNA methyltransferase; na, not available; OS, overall survival; PFS, progression-free survival; pre, preoperative at admission; STR, subtotal resection

## Discussion

Cerebellar-located glioblastoma is a rare entity, occurring only in 0.4–3.4% of all cases of glioblastoma in adults (Babu et al. 2013). The scarcity of data concerning this patient group leads to a significant deficiency in available information. This deficit hinders a comprehensive understanding of both clinical and biological characteristics inherent to this rare tumor entity. Recent published data suggest cerebellar and supratentorial glioblastoma to be characterized by significantly different frequencies of molecular subclasses (Schulte et al. 2020). Zhou et al., for example, revealed an independent role as prognostic factor of *OLIG2* expression for patients with cerebellar glioblastoma (Zhou et al. 2023).

Further molecular analysis revealed that cerebellar glioblastoma themselves comprised a quite heterogeneous, methylation profile-based tumor entity with the so-called AAP subclass (anaplastic astrocytoma with piloid features) among the most frequent (Reinhardt et al. 2019). With regard to topographical, molecular and histopathological characteristics among others, cerebellar glioblastoma might imply quite different long-term outcome pattern than in case of their supratentorial counterparts.

Unfortunately, due to the rare incidence of a cerebellar glioblastoma, existing literature on survival rates of this special entity fails to present any sort of homogenous data pool. In the literature since 2000, there are currently 868 reported cases of cerebellar glioblastoma. However, studies based on cancer registries were excluded from our analysis due to concerns about the accuracy of data regarding tumor localization, the distinction between primary tumors and metastases, treatment details, and survival outcomes. This exclusion was necessary to ensure the reliability and specificity of the data we analyzed. According to our inclusion criteria, the number of cerebellar glioblastoma cases that were eligible for review is 72.

Referred to data collected from 3 up to 14 patients with cerebellar glioblastoma between 1975 and 2011, several previous retrospective studies have reported worse OS for cerebellar glioblastoma compared to supratentorial located glioblastoma (Babu et al. 2013; Gopalakrishnan et al. 2012). In contrast, analyzing a more contemporary single-center experience with 5 patients between 2002 and 2012, Milinkovic et al. in turn described a significant survival advantage of cerebellar glioblastoma patients compared to a pooled cohort of patients with supratentorial glioblastoma with an OS of 18 months (Milinkovic et al. 2014).

Such inconsistent outcome data might partly be ascribed to molecular, histopathological as well as clinical and surgical intrinsic individual features that might lead to a selection bias in a vanishingly low number of patients with cerebellar glioblastoma.

Key factors influencing glioblastoma prognosis include patient age at surgery, KPS at admission, the extent of tumor resection in terms of STR and GTR, MGMT promoter methylation status und IDH-status (Li et al. 2016; McGirt et al. 2009; Radke et al. 2019; Smrdel et al. 2016). In the present analysis, IDH-status did not significantly differ between the groups of patients with cerebellar and supratentorial glioblastoma. This is a crucial point, as the median overall survival for patients with IDH-mutated tumors is reported to be 31 months, significantly longer than the 15-month survival for those with wild-type IDH1 or IDH2 genes (Yan et al. 2009).

Furthermore, advancements in microsurgical techniques and more tailored chemotherapeutic strategies based on MGMT-promoter methylation status over the past 10 to 20 years have influenced survival outcomes. This progress, coupled with the implementation of the Stupp protocol since 2005 (Stupp et al. 2005) as the standard care, has markedly improved survival rates. Therefore, comparing current survival data with those from earlier periods (up to 30–50 years ago) may not accurately reflect the outcomes of patients treated under contemporary standards (Jeswani et al. 2013; Takahashi et al. 2014).

When discussing surgical techniques and the EOR, resection in highly eloquent regions, such as the vermis, cerebellar peduncles, and deep cerebellar nuclei, poses significant risks. These areas are crucial, for example, for motor coordination and cognitive functions, and resection can lead to severe negative outcomes. We were unable to find specific recommendations regarding the surgical management of gliomas extending to these regions, nor can we provide them ourselves.

As highlighted by Gomes et al., surgical damage to cerebellar peduncles and deep cerebellar nuclei leads to extensive white matter changes beyond the cerebellum, including disruption of connections to the cerebello-thalamo-cortical pathways, which are essential for motor control and learning (Gomes et al. 2021). The superior cerebellar peduncle, in particular, serves as the primary efferent pathway from the cerebellum, and damage to it is associated with substantial impairments in motor timing and learning tasks. Additionally, the cerebellar nuclei are critical for both motor output and cognitive processing. Therefore, resection in these regions may result in widespread and long-lasting deficits, making it a less advisable option unless absolutely necessary.

Tumor spread to the cerebellar peduncles often indicates a later stage of the disease, typically associated with brainstem invasion. Consequently, all patients in our study with brainstem infiltration, regardless of whether the tumor was supratentorial or cerebellar, were excluded. In other studies, we found no detailed information on postoperative neurological status or specific preoperative symptoms following resection in the vermis, cerebellar peduncles, or deep cerebellar nuclei.

In our patient series, one patient who underwent GTR in the vermis achieved an OS of 49 months with no unusual disability. However, it remains inconclusive whether tumor spread to the eloquent regions constitutes a contraindication for GTR. Further studies are necessary to assess the long-term outcomes of resection in these regions.

To the best of our knowledge, the present study is the first to apply propensity score matching using established prognostic factors to facilitate a more robust comparative survival analysis between cerebellar and supratentorial glioblastoma patients that had undergone surgical resection starting in 2005. Our findings suggest that survival rates for patients with cerebellar glioblastoma did not differ from those of glioblastomas located in other regions.

In particular, the OS rate of our patient cohort with cerebellar glioblastoma was comparable to the latest available data from other institutions (Milinkovic et al. 2014; Cho et al. 2019; Utsuki et al. 2012).

Further, PFS rates did not significantly differ between these entities of different glioblastoma localizations. Although there are only few publications reporting data on PFS of patient with cerebellar glioblastoma, the acknowledged data appears to be similar to our population (Hong et al. 2018; Picart et al. 2018; Milinkovic et al. 2014; Gopalakrishnan et al. 2012; Utsuki et al. 2012).

In our cohort, we observed one patient with cerebellar glioblastoma (13%) with postoperative hemorrhage resulting in early postoperative death. In the literature, there are limited reports on postoperative complications in surgery for cerebellar glioblastoma, and our systematic review did not reveal any comprehensive analysis regarding complications specifically associated with cerebellar glioblastoma surgery. Nonetheless, there is some sparse data on complications related to posterior fossa surgery, including cerebellar edema, hydrocephalus, cerebellar hematoma, and cerebellar mutism, which we address in the discussion (Dubey et al. 2009).

However, there is a lack of sufficient data from multi-center studies that allows a valid assessment of survival of cerebellar glioblastoma patients especially taking into account possible histological or histochemical heterogeneity.

Further multicenter-based studies are needed to design tailored interdisciplinary modern treatment and aftercare for patients suffering from cerebellar glioblastoma.

### Limitations

The present study has several limitations. The statistical analysis and data collection were retrospective and included only a small cohort from a single institution. Given the rarity of cerebellar glioblastoma, providing Level I evidence with a Grade A recommendation is highly unlikely. Nevertheless, the use of a matched pair approach may help mitigate some confounding factors in comparisons with supratentorial glioblastoma. This approach could justify the conception and establishment of a large-scale, cross-regional database for further analysis of this rare entity. Unfortunately, due to the extended timeframe of data collection, some histological samples from the cerebellar cohort are no longer available, as certain samples have been archived since 2009. As a result, reclassification according to the 2021 WHO criteria for all eight cerebellar glioblastoma samples and further genetic analysis are not feasible within the scope of this study.

## Conclusions

The present findings indicate no significant difference in the prognosis between patients with supratentorial and infratentorial glioblastomas. Current data on cerebellar glioblastomas is notably scarce and predominantly derives from single center series encompassing only a small cohort of patients. The establishment of large-scale, multicenter databases will allow future treatment teams to offer this small population of glioblastoma patients individualized therapy based on strong evidence.

## Electronic supplementary material

Below is the link to the electronic supplementary material.

Supplementary Material 1

## Data Availability

The datasets generated during and/or analyzed during the current study are available from the corresponding author on reasonable request.
