# Supplementary material for: Cerebellar glioblastoma in adults: a comparative single-center matched pair analysis and systematic review of the literature
Source: J Cancer Res Clin Oncol. 2024 Sep 28;150(9):432. doi: 10.1007/s00432-024-05959-0 (PMC11438707; doi:10.1007/s00432-024-05959-0)
Supplement: Supplementary file 1 — Supplementary Material 1 [file 432_2024_5959_MOESM1_ESM.pdf]

PRISMA 2020 flow diagram for new systematic reviews which included searches of databases and registers only

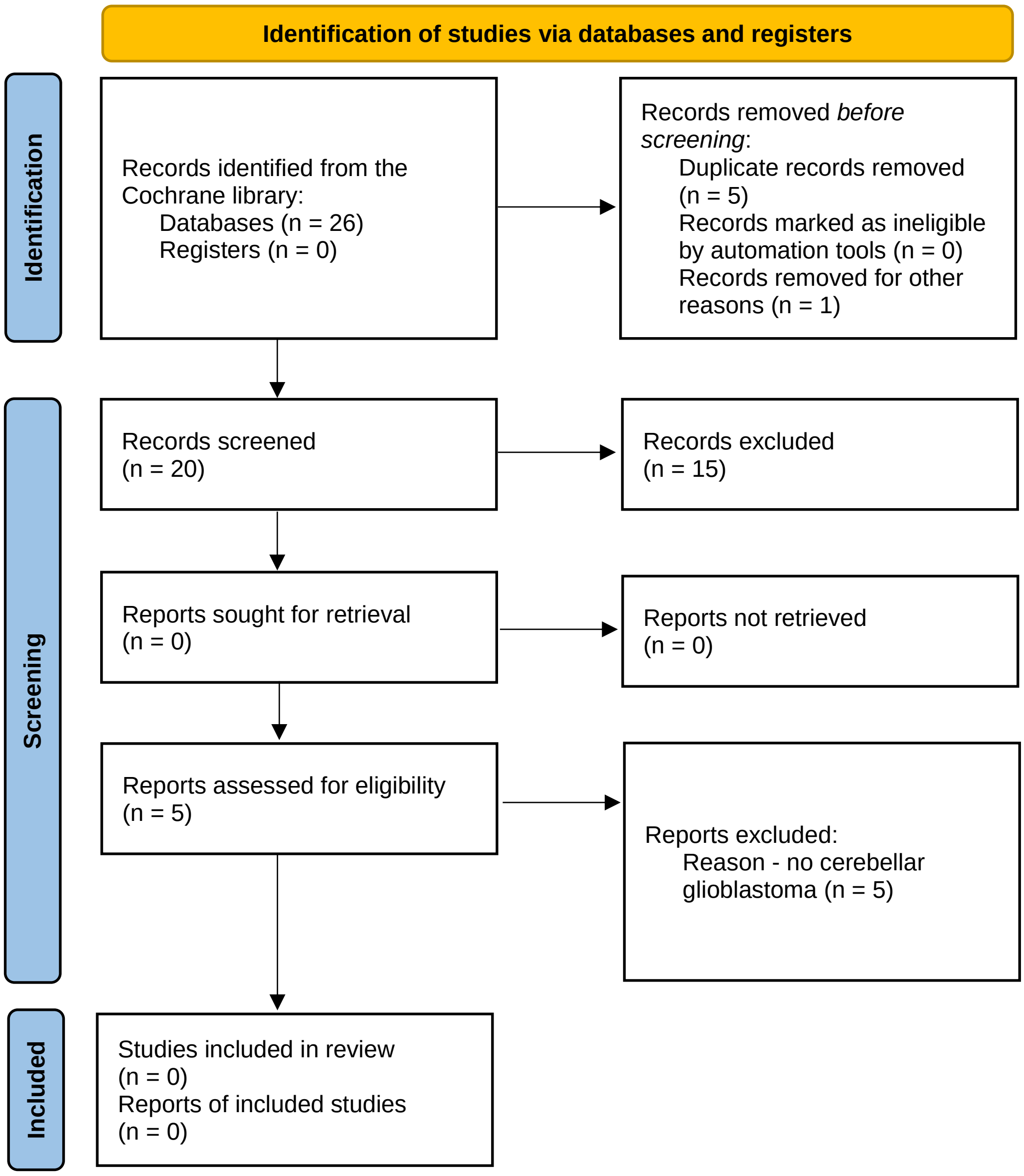

Source: Page MJ, et al. BMJ 2021;372:n71. doi: 10.1136/bmj.n71.

This work is licensed under CC BY 4.0. To view a copy of this license, visit https://creativecommons.org/licenses/by/4.0/
